# The long and the short of it: Salivary telomere length as a candidate biomarker for hypertension and age‐related changes in blood pressure

**DOI:** 10.14814/phy2.15910

**Published:** 2024-01-15

**Authors:** Hollie Speer, Andrew J. McKune, Andrew P. Woodward

**Affiliations:** ^1^ University of Canberra Research Institute for Sport and Exercise (UCRISE), University of Canberra Bruce Australian Capital Territory Australia; ^2^ Discipline of Biokinetics, Exercise and Leisure Sciences, School of Health Science University of KwaZulu‐Natal Durban South Africa; ^3^ Faculty of Health University of Canberra Bruce Australian Capital Territory Australia

**Keywords:** aging, blood pressure, hypertension, telomere length

## Abstract

Hypertension becomes more prevalent with increasing age. Telomere length (TL) has been proposed as a candidate biomarker and can be accessibly extracted from saliva. However, clarity is needed to evaluate the suitability of using TL as a predictor in such instances. This study investigated salivary TL in a cohort of older adults from the 2008 Health and Retirement Study (*n* = 3329; F: 58%, mean age: 69.4, SD: 10.3 years) to examine any associations with blood pressure (BP). A Bayesian robust regression model was fit using weakly informative priors to predict the effects of TL with age, sex, systolic BP (SBP), diastolic BP (DBP), and treatment status. There were small effects of treatment (*β*: −0.07, 95% CrI [−0.33, 0.19], pd: 71.91%) and sex (*β*: −0.10, 95% CrI [−0.27, 0.07], pd: >86.78%). Population effects showed a reduction of 0.01 log_*2*_ units in TL with each year of advancing age (95% CrI [−0.01, −0.00]). Conditional posterior predictions suggest that females, and treated individuals, experience greater change in TL with increasing age. Bayes *R* ^*2*^ was ~2%. TL declines with increasing age, differs between sexes, and appears to be influenced by antihypertensive drugs. Overall, all effects were weak. The data do not currently support the suitability of salivary TL as a biomarker to predict or understand any age‐related changes in BP.

## Graphical abstract

A schematic rationale depicting the contributing roles of aging and oxidative stress in the development of vascular dysfunction and hypertension, presented with a summary of results where salivary telomere length was used as a means to investigate the age‐related changes in blood pressure.

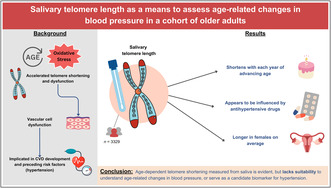

## 1 INTRODUCTION

Vascular aging is associated with various physiological changes at cellular and molecular levels, including oxidative damage and telomere attrition (Emami et al., 2022; Kirkwood, 2005). For this reason, it comes as no surprise that investigations into age‐related conditions often consider changes in these variables to predict (or establish risk parameters for) disease onset and severity. The prevalence of hypertension increases with advancing age and is a strong predictor of future adverse cardiovascular events and cardiovascular disease (CVD) (Roseleur et al., 2022), increasing the risk for ischemic stroke by 38% in females (Gorgui et al., 2014). While in an older population CVD is the leading cause of death, the preceding risk factors are often triggered in the younger years of life but may be delayed in clinical appearance (Emami et al., 2022). Assessment of telomere length (TL) has the capacity to provide potentially useful information relating to the lifespan, and the overall “health status” of a cell which is expected to be poorer in the presence of disease (Shammas, 2011). Physiologically speaking, the length of telomeres should shorten as part of natural human aging to preserve genomic information that could be lost with the gradual reduction in DNA strand length during cellular division (Allsopp et al., 1995; Shammas, 2011). However, inherent telomere shortening can be differentiated from accelerated telomere shortening, where the latter represents an implicit deterioration in function that can be directly related to the aging process and age‐related diseases, including those of the vasculature (Huang et al., 2020; Serra et al., 2003; Spyridopoulos et al., 2009). Telomere shortening has been observed in vascular endothelial cells, smooth muscle cells, and cardiomyocytes during aging (Edo & Andrés, 2005). Current associations between increasing age and cardiovascular mortality have identified population effects of longitudinal increases in systolic blood pressure (SBP) by ~7 mmHg per decade among adults over the age of 40 years (Gurven et al., 2012). The increases in SBP particular to this age demographic may be due to the dysfunction of the vascular endothelium, accompanied with an increase in oxidative distress–either due to intrinsic antioxidant deficiency, or the reduced clearance capacity of oxidants, with advancing age (Kozakiewicz et al., 2019). It has been observed that oxidants induce premature senescence in vascular smooth muscle cells, coupled with accelerated telomere shortening and altered telomerase activity (Cao et al., 2002; Matthews et al., 2006). Age‐associated endothelial dysfunction and disturbances in cellular redox homeostasis often precede the development and onset of hypertension (Minhas et al., 2022). Within the last decade, meta‐analyses (D'Mello et al., 2015; Haycock et al., 2014) and population studies (Yang et al., 2009) have demonstrated inverse associations between leukocyte TL and risk of developing hypertension, independent of conventional vascular risk factors. Meanwhile, another study suggests that there is no difference in mean aortic TL between hypertensive and normotensive individuals (Morgan et al., 2014). Therefore, understanding the factors influencing age‐related changes in BP and the onset of hypertension development is of pertinent clinical relevance due to the stark increase in CVD‐related mortality in later life. Associations between TL more broadly, and the vascular adaptations that occur during and as a result of hypertension onset, have been investigated in previous experimental and human studies (Bhupatiraju et al., 2012; Tellechea & Pirola, 2017). However, the functional relationship between the two, and the suitability of TL as an independent biomarker in general, remains under question. Currently within the literature, TL is more commonly assessed from leukocytes and whole blood, but can also be assessed from nonblood, disease relevant tissue types (i.e., lung, arterial, and skeletal muscle) (Demanelis et al., 2020). In 2020, Demanelis et al. (Demanelis et al., 2020) measured TL across 24 tissue types and showed that while TL is not constant, it is correlated, across tissues. Due to the accessibility and non‐invasiveness of TL extraction from saliva, further investigation is required to assess if the previous associations between leukocyte TL and hypertension can translate to salivary TL, and therefore evaluate its use as a candidate biomarker for hypertension. Age‐dependent associations with telomere shortening have been observed in humans in vivo and in vitro (Allsopp et al., 1995), as well as explored in terms of predicted maximal life expectancy (Steenstrup et al., 2017). Some epidemiological studies (Benetos et al., 2018; Huang et al., 2020; Masi et al., 2014) have examined the association between TL from a multitude of tissue types and hypertension, all with varying methodological approaches and conclusions. Using similar methodological principles to previous studies in the broader field (Elliott‐Sale et al., 2020; Jones et al., 2020), we used a Bayesian approach to conduct analyses of basic demographic information (age and biological sex), BP (systolic and diastolic measurements), hypertensive treatment status (treated or untreated), and salivary TL data extracted from the 2008 Health and Retirement Study (HRS). Understanding the role of biomarkers in circulatory conditions or diseases is pivotal for targeted and early intervention strategies aimed at improving favorable lifestyle outcomes. Therefore, the aim of this analysis was to investigate salivary TL in a cohort of older adults and examine the effects of increasing age and any associated influences on BP measurements, with attempts to critically assess the suitability and utility of using salivary TL as a biomarker for hypertension onset or severity.

## 2 MATERIALS AND METHODS

### 2.1 Data and population

Data were extracted from the HRS 2008 wave. The HRS is sponsored by the National Institute on Aging (grant number NIA U01AG009740) and is conducted by the University of Michigan. The use of this database for epidemiological analyses is well established (Frank and Denis, 2013; Pleiss et al., 2023; Yu et al., 2023). The 2008 Cross‐Wave and Physical Measurement public use datasets were used to obtain demographic and measured information, and the authors were granted access to sensitive telomere data collected from participants in the same year. All data were collated, and participants were matched between datasets using a combination of their de‐identified Household Identification and Person Numbers using Microsoft Excel (Microsoft, v16.0) and imported into R (v4.0.3) (R Core Team, 2020) for cleaning and statistical analysis. The HRS uses a stratified, multistage cluster sample weight designed to represent the US population with respects to age, sex, and race. Informed and written consent were obtained from all participants.

A total of 5808 participants provided buccal samples for TL assessment. Of these participants, 2479 had incomplete data for BP and treatment status and analysis proceeded with list wise deletion (*n* = 3329 included), assuming the data were missing at random (Bhaskaran & Smeeth, 2014).

### 2.2 Blood pressure

Measurement of BP followed standardized protocols and can be found in the 2008 data description files (https://hrs.isr.umich.edu/).

### 2.3 DNA collection and extraction for telomere length assessment

The 2008 telomere data include average TL data from 5808 HRS respondents who consented and provided a saliva sample during the 2008 interview wave. Average TL was measured using quantitative PCR (qPCR) by comparing telomere sequence copy number in each participant's sample (*T*) to a single‐copy gene copy number (*S*). The resulting *T/S* ratio is proportional to a reference mean TL. *T/S* ratio was logarithmically transformed for analyses.

### 2.4 Data analyses and processing

Data were analyzed using the “brms” package (Bürkner, 2017) in R (v4.0.3) (Kruschke & Liddell, 2018; R Core Team, 2020) to model relationships between *T/S* ratio (expressed as TL on log_*2*_ scale), age, diastolic blood pressure (DBP), SBP, and sex, inclusive of an individual's treatment status. Bayesian methods for a linear‐style regression model were utilized, with a Student's‐*t* response distribution (Kruschke, 2013). Inference was by an estimation approach and did not consider hypothesis testing (Kruschke & Liddell, 2018). Model comparisons (Table S1) to evaluate the predictive performance of a generalized additive model using a smooth function of age, and a linear‐style model without a smooth function, were performed using leave‐one‐out cross‐validation (LOO) (Vehtari et al., 2021). There were no statistically influential differences between the models (looic estimates = 2977.8 [SE: 124.5], 2978.8 [SE: 124.4], respectively) meaning that models were equivocal, and therefore the modeling proceeded with the simpler linear‐style approach.

The model can be characterized using the general equation:

$$\log_{2}\left(\mathrm{TL}\right)\sim \left(\beta ₀\right)+\sum \left(\beta_{n}\cdot X_{n}\right)+t\left(0,\sigma ,\nu \right)$$

where: TL, telomere length; *β*₀, intercept; *β*ₙ, coefficient for the nth predictor *X*ₙ; *σ*, scale of the residual error distribution; *ν*, degrees‐of‐freedom (DoF) of the residual error distribution. This allowed for direct estimations of the probability distribution of the treatment effect, which can be interpreted as the probability of different effect sizes given the observed data (Etz & Vandekerckhove, 2018; Kruschke & Liddell, 2018). Prior distributions were intended to be weakly informative (*t‐*distributions with a location of 0, and ν of 3) and were used for all fixed effects, with a scaled parameter set for all continuous variables to correspond to the observed scale of the response variable, so that inference was driven predominantly by the data (Gelman et al., 2008; Lemoine, 2019). Results are presented as posterior means and 95% credible intervals (CrI). Model convergence was assessed with the Rhat statistic, which should be below 1.01 (Vehtari et al., 2021), and effective sample sizes (ESS) above 1000 were considered reliable (Bürkner, 2017), consistent with diagnostic Markov chain Monte Carlo (MCMC) methods used in Bayesian statistics. Following the Sequential Effect eXistence and sIgnificance Testing (SEXIT) framework, indices of effect existence along the probability of direction (pd) were quantified using bayestestR (Makowski et al., 2019). The pd represents the probability (from 50% to > 99%) with which an effect goes in a particular direction (i.e., positive [>0] or negative [<0]).

### 2.5 Visualization of conditional marginal effects

**FIGURE 1:**
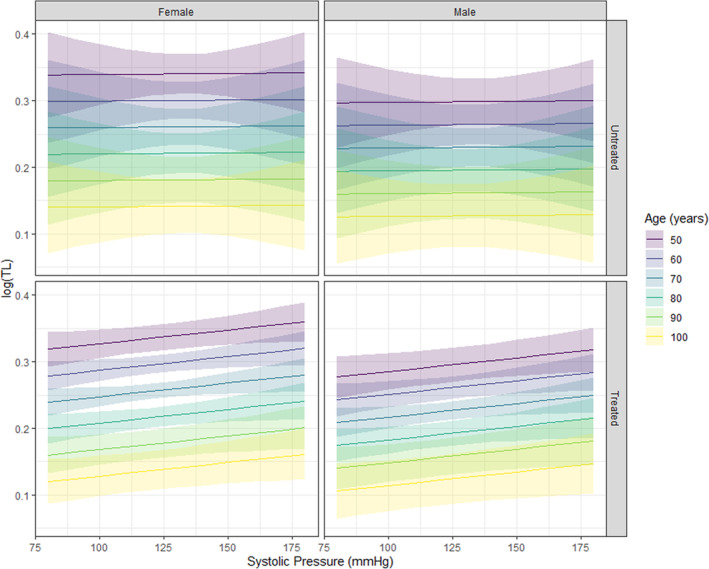
Posterior predictions representing the expected average value of TL with changes in SBP, given all other conditions are true. Females, regardless of treatment, have longer TL on average. In both sexes, TL appears to be longer in treated individuals when pressure exceeds 125 mmHg (shading either side of the line shows 95% uncertainty intervals). Importantly, DBP within the graphic is informed at values set at a pressure of ^2^/_3_ multiplied by that of SBP, to reflect a biologically feasible measurement with any given change in SBP. DBP, diastolic blood pressure; SBP, systolic blood pressure; TL, telomere length.

**FIGURE 2:**
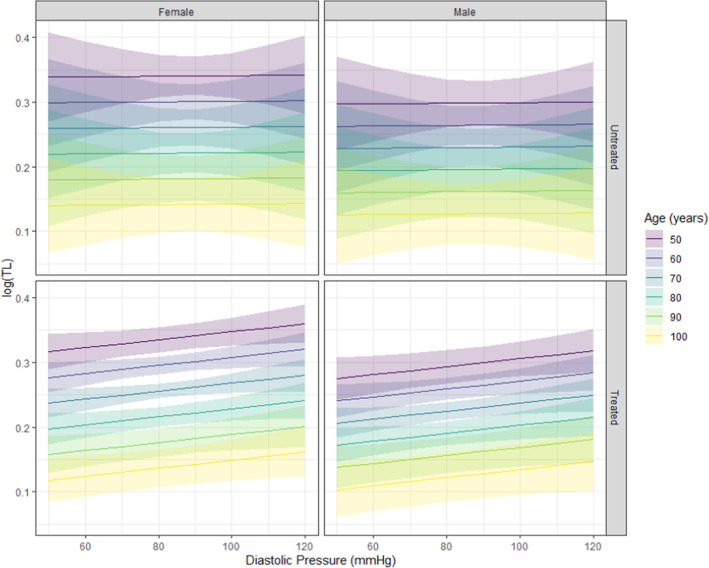
Posterior predictions representing the expected value of TL with changes in DBP, given all other conditions are true. As with Figure 1, females, regardless of treatment, appear to have longer TL on average. In both sexes, TL appears to be longer in treated individuals when DBP increases above 90 mmHg (shading either side of the line shows 95% uncertainty intervals). SBP is informed within the graphic as a constant function of DBP (expressed as DBP + 2/3) to reflect a biologically feasible pressure combinations with any given change in DBP. DBP, diastolic blood pressure; SBP, systolic blood pressure; TL, telomere length.

Estimated age effects conditional on treatment status (treated: 50% of sample) and sex (females: 50% of sample), and their contrasts, were obtained from the fitted estimates of the posterior samples and used to compute expected mean response values of TL, with uncertainty intervals set at 95%. Posterior predictions were posed to visualize the expected mean response value of TL given either systolic (Figure 1) or diastolic (Figure 2) pressure. To represent the expected mean response values across pressures, user‐defined values were set for SBP and DBP, and age was specified in decades from 50 to 100 years (Lüdecke, 2018; Williams, 2012). Graphics were built with ggplot2 (Wickham, 2016).

## 3 RESULTS

### 3.1 Descriptive summary

Participants (*n* = 3329, F: 58.1%, M: 41.9%) were aged between 38 and 100 years (mean: 69.4, SD: 10.3 years) at the time of data collection. Mean SBP was 132 mmHg (SD: 20.3) and mean DBP was 79 mmHg (SD: 11.4). Over 90% of the population were receiving prescribed treatment for hypertension. Mean population TL expressed as *T/S* ratio was 1.38 (SD: 0.72). Medians and interquartile ranges are summarized in Table 1.

**TABLE 1:** Descriptive statistics of participants (*n* = 3329) included in the model, expressed as median and inter‐quartile ranges (IQRs) or count (%).

	Total (*n* = 3329)				
	Min	25%	Median	75%	Max
Age (years)	38	64	71	78	100
SBP (mmHg)	72	121	133	148	223
DBP (mmHg)	43	72	79	87	137
Log_2_ (TL)	−2.32	0.15	0.35	0.58	4.27
TL (*T*/*S* ratio)	0.19	1.11	1.28	1.49	19.30

	Count	Percent (%)
Treated	3016	90.6
Untreated	313	9.40
Female	1937	58.1
Male	1392	41.9

Abbreviations: DBP, diastolic blood pressure; SBP, systolic blood pressure; TL, telomere length.

### 3.2 Model implementation and indices of effect existence

The model accounted for approximately 2% of observed variation (Bayes *R* ^2^ estimate = 0.02, 95% CrI [0.012, 0.024]). Posterior predictive checks showed no evidence of systematic lack‐of‐fit. All estimations successfully converged (Rhat = 1.001) and indices were >1000. Model summaries are shown in Table 2. Point estimates (posterior mean) and 95% CrI of the parameters are reported for coefficients (Table 3). Of note, there were weak independent effects of treatment status (*β*: −0.07, 95% CrI [−0.24, 0.14]) and sex (*β*: −0.10, 95% CrI [−0.18, 0.05]), and extraction of population‐level (“fixed”) effects for age showed an associated decline of 0.01 log_*2*_ units in TL with each year of advancing age (95% CrI [−0.01, −0.00]; Table S2). All other effects were negligible. Statistical summaries for the base model, full model, and general additive model (for model comparisons) can be found in Tables S3–S5, respectively. Probability of direction statements generated for the full model are provided in Table S6.

**TABLE 2:** Summary of the base model (*n* = 5808), representing estimate (mean) and 95% CrI of the *t*‐distribution (residual error).

Parameter	Estimate	Lower 95% Cr level	Upper 95% Cr level
Intercept	0.37	0.26	0.28
Family‐specific parameters			
Scale	0.27	0.26	0.28
DoF	3.25	2.96	3.54
Formula: log_*2*_(TL) ~1			

Abbreviations: CrI, credible intervals; DoF, degrees‐of‐freedom; TL, telomere length.

**TABLE 3:** Parameter estimates from the full model for TL using the parameters of interest (SBP, DBP, treatment status, sex, and age) including interaction terms for age and sex; treatment status and blood pressure, for participants (*n* = 3329).

Parameter	Estimate	Lower 95% Cr level	Upper 95% Cr level
Intercept	0.77	0.50	1.05
SBP	0.00	−0.00	0.00
DBP	−0.00	−0.00	0.00
Treated	−0.07	−0.34	0.18
Untreated	0^a^	‐	‐
Sex: Male	−0.10	−0.26	0.07
Sex: Female	0^a^	‐	‐
Age	−0.01	−0.01	−0.00
Treated × SBP	−0.00	−0.00	0.00
Treated × DBP	0.00	−0.00	0.01
Sex: Male × Age	0.00	−0.00	0.00
Sex: Female × Age	0^a^	‐	‐
Family specific parameters			
Scale	0.27	0.25	0.28
DoF	3.04	2.72	3.41
Bayes *R* ^2^	0.02	0.01	0.02

*Note*: Formula: log_2_ (TL) ~ 1 + SBP × treatment status + DBP × treatment status + Sex × age. Relative performance was assessed using LOO information‐criterion statistic (estimate: 2978.3, SE: 124.5).

^a^ Reference level for dummy‐coded categorical predictor.

Abbreviations: DBP, diastolic blood pressure; DoF, degrees‐of‐freedom; SBP, systolic blood pressure; TL, telomere length.

#### 3.2.1 Treatment status

Sequential effect existence suggests that the effect of being treated (point estimate = −0.07, 95% CrI [−0.33, 0.19]) had a 71.38% probability of being negative (pd < 0).

#### 3.2.2 Biological sex

The effect of male sex (point estimate = −0.10, 95% CrI [−0.27, 0.07]) had an 86.78% probability of being negative (pd < 0).

### 3.3 Estimated effects and approximate posterior probability

As shown in Figures 1 and 2, estimated effects at representative values revealed small effects of sex on TL changes with age. The slope of change in TL with age was different between sexes (posterior mean for males: −0.003, 95% CrI [−0.01, −0.00]; posterior mean for females: 0.001, 95% CrI [−0.00, 0.00]), with an approximate 75% posterior probability of females experiencing a greater change in TL with age, considering estimated age effects conditional on sex and their contrasts (posterior mean: −0.00, 95% CrI [−0.00, 0.00]).

## 4 DISCUSSION

The current study focused on evaluating salivary TL as a predictor for age‐related changes in BP, considering sex and hypertensive treatment status (i.e., treated, or untreated). This was driven by the notion that natural age‐related increases in BP, as well as sex differences in hypertension risk, may influence/be influenced by natural telomere shortening i.e., accelerate telomere attrition (Benetos et al., 2018; Brouilette et al., 2003). Understandably, the sheer biological variability in the parameters evaluated, and the small effects, highlight that in this context, salivary TL lacks utility as a candidate biomarker for hypertension (reflected in the observed model variance: Bayes *R* ^2^ estimate = 0.02, 95% CrI [0.012, 0.024]). Irrespectively, posterior predictive checks demonstrated that the model appeared to be an unbiased description of the data. Our findings support the existing proposition that TL declines with increasing age, and independently suggest that on average, TL is shorter in males than females, and in individuals receiving antihypertensive treatment (in both sexes). Although, these effects were very weak, and without considering any influencing factors are unlikely to be a true representation of real‐life circumstances.

At a cellular level, aging can be observed as the restriction in cellular division capacity prior to entering replicative senescence, which can be driven by oxidative stress, telomere shortening, and many other complex processes (Fyhrquist et al., 2013). Our independent finding of shorter TL (quantified as −0.01 log_*2*_ units) with each year of increasing age is somewhat consistent with existing cross‐sectional studies (Rehkopf et al., 2016; Yu et al., 2023). Interestingly though, the rate of cellular aging (as indicated by shortened TL) particularly in late midlife, has been shown to relate more to vascular damage, independent from other CV risk factors (Masi et al., 2014). A 2016 meta analyses (Tellechea & Pirola, 2017) of 3097 participants (*n* = 1415 hypertensive, *n* = 1682 control) indicated that leukocyte TL may be shorter in hypertensive individuals compared with normotensive individuals, but suggested that studies controlling for confounding effects would be needed to confirm these findings and further explore sources of heterogeneity. A confounding factor we explored in the current study is the effect of hypertensive treatment, and small, independent effects suggested a shorter average TL in those who were treated, compared to untreated (*β*: −0.07, 95% CrI [−0.33, 0.19]). We could speculate that even though an individual may be treated, there could be some irreversible degree of vascular damage that may have already occurred—but a claim like this would need to consider additional factors. To visually explore these independent findings further, we posed reasonable posterior predictions to generate graphics based on a representative form of the full model (fitted estimates), using age, in decades, from 50 to 100 years across a set range of biologically feasible measures of diastolic and systolic pressures, conditional on sex and treatment status (comprised of estimates from females: 50%, treated: 50%). To the contrary, the expected average value of TL appeared to be longer in the treated groups of males and females with increases in SBP beyond ~125 mmHg (Figure 1) and increases in DBP beyond ~90 mmHg (Figure 2). While the figures do not reflect individuals within a clinically “controlled” hypertension range who are treated, and individuals with “masked” hypertension who are untreated, this is an interesting observation considering the clinical significance of these values represent a need for hypertensive intervention. In 2002, Cao et al. (Cao et al., 2002) found telomerase activity to be selectively enhanced in the aortic tissues of genetically hypertensive rats before the onset of hypertension, and that this corresponded with increased proliferation of vascular smooth muscle cells. Consistent with the increase in telomerase activity, TL was increased within the genetically hypertensive rats, and stabilized without progressive shortening (Cao et al., 2002). The authors suggest this reflects that TL maintenance can occur before significant vascular wall remodeling and the onset of hypertension. Although we cannot speculate the same motivation from our findings, this may call for a closer inspection of salivary telomerase activity in conjunction with salivary TL assessment. More recently, Deng et al. (Deng et al., 2022) similarly report that longer TL is related to an increased risk of hypertension (using data from a genome‐wide association study of participants in the UK BioBank). Indeed, these findings conflict with previous evidence, and the lack of consensus among the literature warrants further investigation in order for these claims to be endorsed. Our observations did indicate that there may be a different effect of pressures, dependent on treatment status (effect of being treated on SBP had a 51.62% probability of being negative [pd <0], effect of being treated on DBP had a 66.11% probability of being positive (pd >0)). Existing literature suggests that antihypertensive drugs may influence cell senescence and intracellular oxidative stress (Münzel & Keaney Jr, 2001; Sorriento et al., 2018) potentially altering TL. Oxidative stress (both oxidative eustress and distress) plays an important role in the molecular processes of vascular aging by way of modulating pro‐inflammatory responses, contributing to vessel and endothelial (dys) function, altering calcium homeostasis in vascular cells, as well as autophagy activation in endothelial and vascular smooth muscle cells (Dudinskaya et al., 2020). It has been observed that specific classes of antihypertensive drugs can affect TL through an endothelial nitric oxide synthase (eNOS)‐dependent anti‐senescence effect in human endothelial cells (Hayashi et al., 2014; Zhang et al., 2020). An important consideration to make here is that broader data, including greater diversity of BP as a continuous measurement (not dichotomized), would be helpful in assessing causality and associated mechanistic effects of antihypertensive drugs.

Coefficients for parameter estimates showed sex‐specific differences in TL (*β*: −0.10, 95% CrI [−0.27, 0.07]), but is highly variable. We identified that the slope of change in TL with age for males (posterior mean: −0.00, 95% CrI [−0.01, −0.00]) was different to the slope for females (posterior mean: 0.00, 95% CrI [−0.00, 0.00]), with an approximate 75% posterior probability of females experiencing a greater change in TL with age. Generally speaking, the life expectancy of females is considered to be longer than that of males on average (Hoogendijk et al., 2019), and we could speculate that perhaps a greater longevity of females is associated with a delay in TL shortening, granted our findings cannot strongly explain this. A longitudinal study published in 2019 demonstrated that women had higher total life expectancies but spent more time in poor health compared to men (Hoogendijk et al., 2019). With this considered, perhaps the use of salivary TL as a general biomarker for longevity could be warranted, but there is no reasonable way of translating this measure into relevant information regarding the quality of health of an individual or a population. It has been postulated that genetic factors, as well as estrogen, can mediate (or slow) TL decline (Fyhrquist et al., 2013; Lee et al., 2005). Considering the mean age of the female population in the present study is generalized to be post‐menopausal, estrogen activity (or lack thereof) may not be a driving factor at play, but given the known alterations in vascular function and redox environments by estrogens (Murphy & Kelly, 2011; Xiang et al., 2021), cannot be ruled out. This could conceive the notion that the varying contribution of sex‐specific hormones may be factors in either determining longer average TL in the female sex at birth, or in the maintenance of TL over the lifetime, independent of other factors. With these observations in mind, it could be warranted to further consider the physiological influence of sex differences, age and hormonal status in the clinical management and control of hypertension (Ahmed et al., 2019).

## 5 STUDY LIMITATIONS AND STRENGTHS

The HRS obtains TL from salivary DNA samples which, while accessible, could be highly variable when self‐collected. Additionally, although we accounted for treatment status overall, a limitation to this is that the data collected did not discern between the name or class of the antihypertensive drugs, or the prescribed dosage nor duration of treatment, and we did not account for additional medications being taken as potential confounders/interaction effects in the analysis. A criticism of previous population‐based, epidemiological, or cross‐sectional studies assessing TL is that there is currently no known range or value to provide a substantive understanding of what classifies telomeres as “short” or “long”, and this, in part, may be the reason some values are perceived as extreme outliers, which can often be identified and removed from analysis, or arbitrarily classified as shorter or longer based on the mean of a sample. A strength of the current study is that the estimated DoF from the conditional distribution (*ν*: 3.25, 95% CrI [2.96, 3.54]) provides empirical evidence that kurtosis is important for this variable in the observed data, which would otherwise be missed in a model using a normal distribution. Therefore, we propose that exploring some of the more “extreme” outliers to understand more about telomere maintenance and biology, why they appeared and whether it is likely similar values will continue to appear, may be necessary to establish a “homeostatic range” of typical TL distribution.

## 6 FUTURE DIRECTIONS

Where data is available, future longitudinal, cohort studies are warranted to assess the link between the rate of telomere attrition, BP, and antihypertensive treatment—including medication type, duration of treatment, and dosage. In doing so, there is capacity to explore if telomere attrition is inherently maintained over time, or if it occurs at an increased rate during disease development. It would be worth exploring the changes in response to treatment, considering age and sex, and if alternate interventions such as lifestyle modification can support longer TL more effectively at different ages and between sexes. For acute, and more rapid assessments of potential pre‐manifesting vascular mediators, future avenues should investigate more clinically relevant biomarkers or measures. Perhaps, greater efforts should be directed into observing more sensitive changes at cellular and molecular levels in vivo, to detect the manifestation of vessel dysfunction that leads to age‐related changes in BP.

## 7 CONCLUSIONS

While measuring TL can provide valuable insight into the possible role of TL in the pathophysiology of vascular aging and disease, telomere dynamics are highly variable and are not a static property, meaning much more information is needed to understand telomere function and any associated effects on disease onset or severity. Taken together, the overall findings of this study support an age‐dependent association of salivary TL on advancing age, and that there are sex‐specific TL dynamics that may contribute to the development and onset of hypertension. Additionally, we identify that the use of antihypertensive drugs warrants further exploration in relation to vascular telomere activity. However, given the very weak relationships identified in the current analysis, salivary TL cannot provide the required sensitivity to predict or understand any age‐related changes in BP, and the small effects allude to any age‐related changes in BP more likely being explained by other factors. As a candidate biomarker for hypertension, a once‐off assessment of salivary TL is a biological measurement with limited utility.

## AUTHOR CONTRIBUTIONS

A.J.M and H.S conceived the study idea. A.P.W and H.S developed the statistical modeling methods. H.S extracted and collated data and ran preliminary and exploratory analyses. H.S and A.P.W implemented the formal analyses. H.S drafted the manuscript. A.J.M and A.P.W provided critical feedback. All authors revised the manuscript. All authors contributed significantly to the development of the manuscript and approved the final version submitted for publication.

## FUNDING INFORMATION

The authors received no financial support for the research or authorship for this article.

## ETHICS STATEMENT

The HRS obtained informedand written consent from all participants.

## Supporting information


Table S1.
Table S2.
Table S3.
Table S4.
Table S5.
Table S6.

## Data Availability

HRS public data products may be used without restriction; however, distribution of these products is managed by the study themselves. Since HRS conditions of use prohibit redistribution of any HRS data product, all HRS public datasets are considered to be proprietary. The data underlying this article are available via the HRS file download site (https://ssl.isr.umich.edu/hrs/start.php), produced and distributed by the University of Michigan with funding from the National Institute on Aging (grant number NIA U01AG009740).
