# Supplementary material for: The long and the short of it: Salivary telomere length as a candidate biomarker for hypertension and age‐related changes in blood pressure
Source: Physiol Rep. 2024 Jan 15;12(1):e15910. doi: 10.14814/phy2.15910 (PMC10789652; doi:10.14814/phy2.15910)
Supplement: Supplementary file 1 — Table S1. Table S2. Table S3. Table S4. Table S5. Table S6. [file PHY2-12-e15910-s001.docx]

**Supplemental Material**

**Table S1.** Model comparison of a general additive model (GAM) and linear-style regression model using LOO informative-criterion statistic (looic). Model estimates suggest no significant difference, therefore modelling proceeded with a linear style approach

| **GAM** | | | **Linear-style regression model** | | |
| --- | --- | --- | --- | --- | --- |
|  | **Estimate** | **SE** |  | **Estimate** | **SE** |
| **elpd_loo** | -1488.9 | 62.2 | **elpd_loo** | -1489.2 | 62.2 |
| **p_loo** | 12.1 | 0.3 | **p_loo** | 10.7 | 0.2 |
| **looic** | 2977.8 | 124.3 | **looic** | 2978.3 | 124.4 |
| Formula: log_2_(TL) ~ 1 + SYSAVE * TREATMENT_STATUS_cleaned + DIAAVE * TREATMENT_STATUS_cleaned + SEX_CODE + s(AGE) | | | Formula: log_2_(TL) ~ 1 + SYSAVE * TREATMENT_STATUS_cleaned + DIAAVE * TREATMENT_STATUS_cleaned + SEX_CODE * AGE | | |
| Computed from 8000 by 3329 log-likelihood matrix  Monte Carlo SE of elpd_loo is 0.0.  All Pareto k estimates are good (k < 0.5). | | | | | |

**Table S2**. Extraction of population-level estimates using fixef() brms

| **Parameter** | **Estimate** | **Est.Error** | **Q2.5** | **Q97.5** |
| --- | --- | --- | --- | --- |
| **Intercept** | 0.78 | 0.14 | 0.50 | 1.03 |
| **SYSAVE** | 0.00 | 0.00 | -0.00 | 0.00 |
| **TREATMENT_STATUS_cleaned Treated** | -0.07 | 0.13 | -0.33 | 0.19 |
| **DIAAVE** | -0.00 | 0.00 | -0.00 | 0.00 |
| **SEX_CODEMale** | -0.10 | 0.09 | -0.26 | 0.07 |
| **AGE** | -0.01 | 0.00 | -0.01 | -0.00 |
| **SYSAVE:TREATMENT_STATUS_cleaned Treated** | -0.00 | 0.00 | -0.00 | 0.00 |
| **TREATMENT_STATUS_cleanedTreated: DIAAVE** | 0.00 | 0.00 | -0.00 | 0.01 |
| **SEX_CODEMale:AGE** | 0.00 | 0.00 | -0.00 | 0.00 |

**Table S3.** Full output summary for *t-*distribution

| **Population-Level Effects:** | **Estimate** | **Est.Error** | **l-95%CI** | **u-95%CI** | **Rhat** | **Bulk_ESS** | **Tail_ESS** |
| --- | --- | --- | --- | --- | --- | --- | --- |
| **Intercept** | 0.37 | 0.00 | 0.36 | 0.37 | 1 | 3124 | 2749 |
| **Family Specific Parameters:** | **Estimate** | **Est.Error** | **l-95%CI** | **u-95%CI** | **Rhat** | **Bulk_ESS** | **Tail_ESS** |
| **sigma** | 0.27 | 0.00 | 0.26 | 0.28 | 1 | 1664 | 2261 |
| **nu** | 3.25 | 0.14 | 2.99 | 3.56 | 1 | 1750 | 2128 |
| Draws were sampled using sampling(NUTS). For each parameter, Bulk_ESS and Tail_ESS are effective sample size measures, and Rhat is the potential scale reduction factor on split chains (at convergence, Rhat = 1). | | | | | | | |
| Computed from 4000 by 5808 log-likelihood matrix  Monte Carlo SE of elpd_loo is 0.0.  All Pareto k estimates are good (k < 0.5). | | | | | | | |
| Family: student  Links: mu = identity; sigma = identity; nu = identity  Formula: log_2_(TL) ~ 1  Data: X2022_BP_TL_DATA_MAY (Number of observations: 5808)  Draws: 4 chains, each with iter = 2000; warmup = 1000; thin = 1; total post-warmup draws = 4000 | | | | | | | |

**Table S4.** Independent output for linear-style regression model, also used for model comparisons

| **Population-Level Effects:** | **Estimate** | **Est.Error** | **l-95%** | **u-95%** | **Rhat** | **Bulk_ESS** | **Tail_ESS** |
| --- | --- | --- | --- | --- | --- | --- | --- |
| **Intercept** | 0.77 | 0.14 | 0.50 | 1.04 | 1 | 6403 | 6000 |
| **SYSAVE** | 0.00 | 0.00 | -0.00 | 0.00 | 1 | 6374 | 5188 |
| **TREATMENT_STATUS_ cleanedTreated** | -0.07 | 0.13 | -0.33 | 0.19 | 1 | 5996 | 5262 |
| **DIAAVE** | -0.00 | 0.00 | -0.00 | 0.00 | 1 | 5599 | 5509 |
| **SEX_CODEMale** | -0.10 | 0.09 | -0.27 | 0.07 | 1 | 5821 | 4644 |
| **AGE** | -0.01 | 0.00 | -0.01 | 0.00 | 1 | 7149 | 6417 |
| **SYSAVE:TREATMENT_ STATUS_cleanedTreated** | -0.00 | 0.00 | -0.00 | 0.00 | 1 | 6437 | 5587 |
| **TREATMENT_STATUS_ cleanedTreated:DIAAVE** | 0.00 | 0.00 | -0.00 | 0.00 | 1 | 5644 | 5452 |
| **SEX_CODEMale:AGE** | 0.00 | 0.00 | -0.00 | 0.00 | 1 | 5782 | 4723 |
| **Family Specific Parameters:** | **Estimate** | **Est.Error** | **l-95%** | **u-95%** | **Rhat** | **Bulk_ESS** | **Tail_ESS** |
| **sigma** | 0.27 | 0.01 | 0.25 | 0.28 | 1 | 5426 | 4764 |
| **nu** | 3.04 | 0.18 | 2.71 | 3.40 | 1 | 5364 | 4859 |
| Draws were sampled using sampling(NUTS). For each parameter, Bulk_ESS and Tail_ESS are effective sample size measures, and Rhat is the potential scale reduction factor on split chains (at convergence, Rhat = 1).  Bayes R^2 value: estimate = 0.02 (Est. Error: 0.00, [Q2.5, 0.01 – Q97.5, 0.02]) | | | | | | | |
| Family: student  Links: mu = identity; sigma = identity; nu = identity  Formula: log_2_(TL) ~ 1 + SYSAVE * TREATMENT_STATUS_cleaned + DIAAVE * TREATMENT_STATUS_cleaned + SEX_CODE * AGE  Data: X2022_BP_TL_DATA_MAY (Number of observations: 3329)  Draws: 4 chains, each with iter = 4000; warmup = 2000; thin = 1; total post-warmup draws = 8000 | | | | | | | |

**Table S5.** Independent output for GAM (smooth function for age), used for model comparisons

| **Smooth terms:** | **Estimate** | **Est.Error** | **l-95%** | **u-95%** | **Rhat** | **Bulk_ESS** | **Tail_ESS** |
| --- | --- | --- | --- | --- | --- | --- | --- |
| **sds(sAGE_1)** | 0.19 | 0.17 | 0.01 | 0.62 | 1 | 2032 | 3411 |
| **Population-Level Effects:** | Estimate | Est.Error | l-95% | u-95% | Rhat | Bulk_ESS | Tail_ESS |
| **Intercept** | 0.36 | 0.13 | 0.11 | 0.62 | 1 | 7584 | 6110 |
| **SYSAVE** | 0.00 | 0.00 | -0.00 | 0.00 | 1 | 7997 | 5436 |
| **TREATMENT_STATUS_ cleanedTreated** | -0.07 | 0.14 | -0.34 | 0.20 | 1 | 7402 | 6066 |
| **DIAAVE** | -0.00 | 0.00 | -0.00 | 0.00 | 1 | 6660 | 5237 |
| **SEX_CODEMale** | -0.04 | 0.01 | -0.07 | -0.02 | 1 | 9479 | 5978 |
| **SYSAVE: TREATMENT_STATUS_ cleanedTreated** | 0.00 | 0.00 | -0.00 | 0.00 | 1 | 8134 | 6008 |
| **TREATMENT_STATUS_ cleanedTreated:DIAAVE** | 0.00 | 0.00 | -0.00 | 0.00 | 1 | 6589 | 5406 |
| **sAGE_1** | -0.22 | 0.52 | -0.95 | 1.15 | 1 | 2755 | 3221 |
| **Family Specific Parameters:** | Estimate | Est.Error | l-95% | u-95% | Rhat | Bulk_ESS | Tail_ESS |
| **sigma** | 0.27 | 0.01 | 0.25 | 0.28 | 1 | 6850 | 6110 |
| **nu** | 3.05 | 0.18 | 2.72 | 3.41 | 1 | 6657 | 6414 |
| Draws were sampled using sampling(NUTS). For each parameter, Bulk_ESS and Tail_ESS are effective sample size measures, and Rhat is the potential scale reduction factor on split chains (at convergence, Rhat = 1).  Bayes R^2 value: estimate = 0.02 (Est. Error: 0.00, [Q2.5, 0.01 – Q97.5, 0.03]) | | | | | | | |
| Family: student  Links: mu = identity; sigma = identity; nu = identity  Formula: log_2_(TL) ~ 1 + SYSAVE * TREATMENT_STATUS_cleaned + DIAAVE * TREATMENT_STATUS_cleaned + SEX_CODE + s(AGE)  Data: X2022_BP_TL_DATA_MAY (Number of observations: 3329)  Draws: 4 chains, each with iter = 4000; warmup = 2000; thin = 1; total post-warmup draws = 8000 | | | | | | | |

**Table S6.** Probability of direction statements for full model summary

| **Parameter** | **pd** |
| --- | --- |
| (Intercept) | 100% |
| SYSAVE | 71.91% |
| TREATMENT_STATUS_cleanedTreated | 71.38% |
| DIAAVE | 68.30% |
| SEX_CODEMale | 86.78% |
| AGE | 100% |
| SYSAVE:TREATMENT_STATUS_cleanedTreated | 51.62% |
| TREATMENT_STATUS_cleanedTreated:DIAAVE | 66.11% |
| SEX_CODEMale:AGE | 73.71% |
